# Indicators of intensive care unit capacity strain: a systematic review

**DOI:** 10.1186/s13054-018-1975-3

**Published:** 2018-03-27

**Authors:** Oleksa G. Rewa, Henry T. Stelfox, Armann Ingolfsson, David A. Zygun, Robin Featherstone, Dawn Opgenorth, Sean M. Bagshaw

**Affiliations:** 1 Department of Critical Care Medicine, Faculty of Medicine and Dentistry, University of Alberta, 2-124 Clinical Sciences Building, 8440 – 112th Street, Edmonton, AB T6G 2B7 Canada; 2 School of Public Health, University of Alberta, Edmonton, AB Canada; 3 Critical Care Strategic Clinical Network, Alberta Health Services, Edmonton, AB Canada; 4 Department of Critical Care Medicine, Cumming School of Medicine, University of Calgary, Calgary, AB Canada; 5 Department of Community Health Sciences, Cumming School of Medicine, University of Calgary, Calgary, AB Canada; 6 O’Brien Institute for Public Health, Cumming School of Medicine, University of Calgary, Calgary, AB Canada; 7 Alberta School of Business, University of Alberta, Edmonton, AB Canada; 8 Alberta Research Center for Health Evidence (ARCHE), Department of Pediatrics, University of Alberta, Edmonton, AB Canada

**Keywords:** Quality, Safety, Adverse event, Indicator, Performance, Capacity, Strain, Organization, Intensive care unit

## Abstract

**Background:**

Strained intensive care unit (ICU) capacity represents a fundamental supply-demand mismatch in ICU resources. Strain is likely to be influenced by a range of factors; however, there has been no systematic evaluation of the spectrum of measures that may indicate strain on ICU capacity.

**Methods:**

We performed a systematic review to identify indicators of strained capacity. A comprehensive peer-reviewed search of MEDLINE, EMBASE, CINAHL, Cochrane Library, and Web of Science Core Collection was performed along with selected grey literature sources. We included studies published in English after 1990. We included studies that: (1) focused on ICU settings; (2) included description of a quality or performance measure; and (3) described strained capacity. Retrieved studies were screened, selected and extracted in duplicate. Quality was assessed using the Newcastle-Ottawa Quality Assessment Scale (NOS). Analysis was descriptive.

**Results:**

Of 5297 studies identified in our search; 51 fulfilled eligibility. Most were cohort studies (*n* = 39; 76.5%), five (9.8%) were case-control, three (5.8%) were cross-sectional, two (3.9%) were modeling studies, one (2%) was a correlational study, and one (2%) was a quality improvement project. Most observational studies were high quality. Sixteen measures designed to indicate strain were identified 110 times, and classified as structure (*n* = 4, 25%), process (*n* = 7, 44%) and outcome (*n* = 5, 31%) indicators, respectively. The most commonly identified indicators of strain were ICU acuity (*n* = 21; 19.1% [process]), ICU readmission (*n* = 18; 16.4% [outcome]), after-hours discharge (*n* = 15; 13.6% [process]) and ICU census (*n* = 13; 11.8% [structure]). There was substantial heterogeneity in the operational definitions used to define strain indicators across studies.

**Conclusions:**

We identified and characterized 16 indicators of strained ICU capacity across the spectrum of healthcare quality domains. Future work should aim to evaluate their implementation into practice and assess their value for evaluating strategies to mitigate strain.

**Systematic review registration:**

This systematic review was registered at PROSPERO (March 27, 2015; CRD42015017931).

**Electronic supplementary material:**

The online version of this article (10.1186/s13054-018-1975-3) contains supplementary material, which is available to authorized users.

## Background

Strained intensive care unit (ICU) capacity is conceptually defined as a discrepancy between the availability of ICU resources and demand to admit and provide high-quality care for patients with critical illness [1, 2]. ICU capacity strain is perceived to contribute to suboptimal care and may modify patient susceptibility to adverse events [3–5]. Strained capacity may influence clinician behavior and alter patient care processes [6, 7]. Recent observations have suggested sustained strain may have negative consequences for ICU clinician wellbeing and the workplace environment [8].

Strained capacity is perceived among ICU professionals to be encountered more frequently due to growing demand for and relatively fixed supply of critical care services [1]. Moreover, strained capacity is perceived to contribute to inefficient healthcare resource use and to negatively impact the satisfaction that patients and families have with the healthcare they receive [1].

Strained ICU capacity is complex and likely influenced by a spectrum of patient-related, health care professional-related and health system-related factors. While selected indicators of capacity strain are well-described and are commonly used by healthcare systems (e.g., census, acuity, new admissions [9, 10]); there has been no systematic interrogation of the literature to define the spectrum of indicators that may inform whether an ICU is experiencing strain.

Currently, there are few robust or validated indicators that quantify the immediate or temporal “stress” an ICU experiences due to strained capacity. Accordingly, we performed a systematic review and evidence synthesis to identify and characterize available indicators of strained ICU capacity. We believe this is an important initial step to develop evidence-informed indicators of strained capacity that may be implemented into routine practice to guide clinical care and policy.

## Methods

We performed a systematic review using methodological approaches recommended in the Cochrane Handbook for Systematic Review of Interventions and described according to the PRISMA-P guidelines (Additional file 1) [11]. Health research ethics board approval was not required for this study. This systematic review was registered at PROSPERO (March 27, 2015; CRD42015017931) [12].

### Systematic review objectives

The core aim of this review was to systematically evaluate the literature to identify proposed indicators of ICU capacity strain. The specific objectives included: (1) to generate an inventory of quality and performance indicators associated with strain on ICU capacity; and (2) to categorize these indicators of ICU capacity strain at the patient-level, ICU-level, and health system-level across attributes of quality indicators (i.e., importance; scientific acceptability; usability; feasibility) and the Donabedian framework (i.e., structure – where healthcare is delivered; process – how healthcare is delivered; outcome – the effects of the delivery of healthcare).

### Search strategy for identification of studies

We developed a comprehensive search strategy in consultation with a research librarian (RF) that was peer-reviewed by a second research librarian [13]. We searched the following electronic databases between August 11 and 24, 2015: Ovid MEDLINE (1946-), Ovid EMBASE (1988-), CINAHL Plus with Full Text via EBSCOhost (1937-), the Cochrane Library (inception-), including the Cochrane Database of Systematic Reviews, the Cochrane Central Register of Controlled Trials (CENTRAL), and Web of Science Core Collection (1900-). We ran update searches in MEDLINE and EMBASE on February 1, 2017. Our search strategy combined the following concepts: (1) intensive care, critical care, critical illness, multi-organ dysfunction, multi-organ failure; (2) quality indicator, quality measure, performance indicator, quality improvement, quality assurance, quality control, performance improvement, best practice, processes of care, complications, adverse event, medication error, safety, effectiveness, efficiency, appropriateness, outcomes assessment, outcome, audit; and (3) strain, capacity, occupancy, census, resource, operations management, acuity, rationing, queuing, avoidable, unplanned, readmission, nighttime, discharge, absenteeism, burnout, workload, discrete event simulation (Additional file 2). Grey literature sources were searched for operations management reports, and selected conference proceedings (Additional file 3). Bibliographic records were exported to an EndNote X7 (Thomson Reuters, Philadelphia, PA, USA) database for screening.

Studies were included if they mentioned all of the following themes: (1) intensive care (i.e., intended to refer to patients (adults, children, and neonates) who are critically ill or at risk for an acute clinical deterioration that may necessitate support in an ICU setting); (2) quality or performance indicator (i.e., any measurable variable intended to evaluate the structure, process, or outcome of care provided to patients); (3) capacity strain (i.e., any measurable variable intended to evaluate the untoward impact at the patient-level, ICU-level, or heath-system level stress on ICU capacity due to the mismatch in demand and supply in our healthcare system). We considered studies published in English and after 1990. Finally, selected levels of evidence included all studies types (i.e., abstracts and full texts) reporting original primary and/or secondary data, as well as administrative reports to government or healthcare agencies.

We used a two-stage process for study selection [14]. First, two reviewers (OGR and SMB) independently screened the titles and abstracts (when available) of search results to determine whether a study fulfilled the general inclusion criteria. Disagreements were resolved by discussion. The full-text versions of all citations classified as “include” by either reviewer were retrieved for in-depth review. The same two reviewers (OGR and SMB independently assessed the eligibility of each full-text manuscript for final inclusion into the review. Any disagreement was resolved by discussion.

### Data abstraction

Two independent reviewers (OGR and SMB) extracted data using standardized, piloted, data extraction report forms. All strain indicators were identified, abstracted, and agreed upon by the two independent authors (OGR and SMB). The following data were abstracted from each citation: author identification, year of publication, title, journal of publication, study design, identified strain indicator(s), along with the operation definition and the relevance of each strain indicator.

Each strain indicator was characterized on its importance, scientific acceptability, usability and feasibility, as similarly performed previously by the authors [14]. Initially, 20% of retrieved citations (*n* = 20) had strain indicators described in duplicate to ensure consistency. Due to significant redundancy in strain indicators, the remainder of citations was extracted by a single reviewed (OGR) [14]. If there was uncertainty, strain indicators were reviewed in duplicate and consensus on its characteristics were achieved through discussion. Each strain indicator was stratified (yes/no) according to whether study authors described it as being importance (i.e., ICU quality, patient-centeredness, healthcare costs); described its scientific basis and rationale; and whether it was operationally usable and feasible (i.e., easy to indicator or implement; able to be integrated into an electronic health record [EHR]).

### Internal validity and risk of bias assessment

We assessed the internal validity of included studies using the Newcastle-Ottawa Quality Assessment Scale (NOS), where studies are scored 0 to 9 based on quality [15]. Studies were rated high quality if they had a total score of 6–9, moderate quality with a score of 4–5, and poor quality if they had a score of 3 or fewer [14].

### Data analysis

The primary analysis was descriptive. Strain indicators were categorized according to the Donabedian framework by stratifying whether each indicator represented a structure, process, or outcome related to ICU capacity strain [16]. Strain indicators were further evaluated by OGR and SMB using the four criteria proposed by the US Strategic Framework Board for a National Quality Measurement and Reporting System, as outlined above [17].

## Results

### Search results

**Fig. 1:**
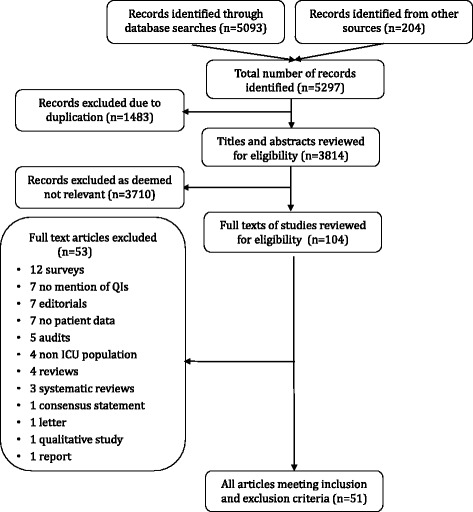
PRISMA flow diagram of retrieved and included records. This flow diagram depicts the identified citations from the medical and grey literature. Of the 54 articles meeting inclusion and exclusion criteria, 40 were full-text articles while 14 were only available in abstract form

Our initial search strategy identified 5297 citations, of which 51 articles satisfied eligibility criteria and were included (Fig. 1). Of these, 40 were full-text articles and 11 were presented in abstract form only (Additional File 4). The majority were cohort studies (*n* = 39; 76.5%), five (9.8%) were case-control, three (5.8%) were cross-sectional, two (3.9%) were modeling studies, one (2%) was a correlational study, and one (2%) was a quality improvement project (Table 1). Most studies were research in nature (*n* = 43, 84.3%), while the remainder where quality improvement projects (*n* = 8, 15.7%).

**Table 1:** Baseline characteristics of included trials

Author	Source	Intent	Design	Population	(*n*)	Strain Measure
Ahmed [1]	Abstract	Quality	Case-control	Adult	161	After-hours discharge queuing
Ahrens [2]	Abstract	Research	Cohort	Pediatric	764	ICU census
Al-Jaghbeer [3]	Full text	Quality	Cohort	Adult	136	After-hours discharge ICU readmission
Amaravadi [4]	Full text	Research	Cohort	Adult	366	Nurse-to-patient ratio
Aytekin [5]	Full text	Research	Correlational	Neonatal	80	Burnout
Azevedo [6]	Abstract	Research	Cohort	Adult	1329	ICU acuity ICU readmission
Beck [7]	Full text	Research	Cohort	Adult	1654	After-hours discharge ICU acuity
Brown [8]	Full text	Research	Cohort	Adult	268,824	ICU readmission
Brown [9]	Full text	Research	Cohort	Adult	214,692	ICU readmission
Chalfin [10]	Full text	Research	Cross-sectional	Adult	50,322	Queuing
Cooper [11]	Full tText	Research	Cohort	Adult	103,984	ICU acuity ICU readmission
Czaja [12]	Full text	Research	Cohort	Pediatric	111,923	After-hours discharge ICU readmission
Dara [13]	Full text	Research	Cohort	Adult	2492	ICU acuity Standardized mortality rate
Duke [14]	Full text	Research	Cohort	Adult	697	After-hours discharge
Duke [15]	Full text	Research	Cross-sectional	Adult	24,935	ICU readmission ICU transfer Queuing Surgery cancellation
Duke [16]	Full text	Research	Cohort	Adult	3004	ICU census ICU refusal Surgery cancellation
Frankel [17]	Full text	Quality	Cohort	Adult	4956	ICU readmission
Frisho-Lima [18]	Full text	Research	Cohort	Adult	127	ICU census
Gajic [19]	Full text	Research	Cohort	Adult	1131	ICU acuity ICU readmission
Gantner [20]	Full text	Research	Cohort	Adult	109,384	After-hours discharge ICU readmission
Goldfrad [21]	Full text	Research	Case-control	Adult	2269	After-hours discharge
Gopal [22]	Abstract	Research	Case-control	Adult	1257	After-hours discharge ICU readmission
Harris [23]	Abstract	Research	Cohort	Adult	13,086	Queuing
Heneghan [6]	Abstract	Research	Cohort	Pediatric	373	ICU readmission
Hung [24]	Full text	Research	Cohort	Adult	1242	ICU acuity Queuing
Iwashyna [25]	Full text	Research	Case-control	Adult	200,499	ICU acuity ICU census
Joynt [26]	Full text	Research	Cohort	Adult	624	ICU census Standardized mortality rate
Kramer [27]	Full text	Research	Cohort	Adult	369,129	ICU acuity ICU readmission
Laupland [28]	Full text	Research	Cohort	Adult	7380	After-hours discharge ICU acuity
Leary [29]	Full text	Quality	Modeling	Adult	3101	ICU census Standardized mortality rate Workload
Lim [30]	Full text	Research	Cohort	Adult	70	ICU acuity
Liu [31]	Full text	Research	Case-control	Adult	6369	ICU acuity
Louriz [32]	Full text	Research	Cohort	Adult	398	ICU acuity ICU census
Nathanson [33]	Full text	Research	Cohort	Adult	124,855	ICU acuity
Parker [34]	Abstract	Research	Cohort	Adult	255	Queuing
Pozzesseres [6]	Abstract	Research	Cohort	Adult	210	Queuing
Priestap [35]	Full text	Research	Cohort	Adult	47,062	After-hours discharge ICU acuity
Pronovost [36]	Full text	Research	Cohort	Adult	2982	Daily rounds by intensivist Nurse-to-patient ratio
Rosenberg [37]	Full text	Research	Cohort	Adult	4208	ICU acuity ICU readmission Standardized mortality rate
Ruse [38]	Abstract	Quality	Cohort	Adult	Unknown	After-hours discharge ICU acuity Queuing
Santamaria [39]	Full text	Research	Cohort	Adult	10,221	After-hours discharge ICU acuity
Singh [40]	Full text	Research	Cohort	Adult	2300	After-hours discharge ICU acuity ICU readmission
Stelfox [6]	Abstract	Research	Cohort	Adult	32,234	ICU readmission
Tobin [41]	Abstract	Research	Cohort	Adult	10,903	After-hours discharge Early ICU discharge ICU acuity Queuing
Town [42]	Full text	Research	Cohort	Adult	60,355	ICU census ICU readmission Turnover Workload
Tucker [43]	Full text	Research	Cohort	Neonatal	13,334	ICU census Nurse-to-patient ratio Turnover
Wagner [44]	Full text	Quality	Cohort	Adult	200,730	ICU acuity ICU census ICU readmission Turnover
West [45]	Full text	Research	Cross-sectional	Adult	38,165	ICU census Nurse-to-patient ratio Turnover Workload
Yergens [38]	Abstract	Research	Cohort	Adult	1770	ICU census
Amarasigham [46]	Full text	Quality	Quality Improvement	Adult	Unknown	Queuing
Barado [47]	Abstract	Quality	Modeling	Adult	6300	Early ICU discharge ICU refusal

The characteristics of the included studies are included above. A full reference of included studies is included in Additional file 4.

*ICU*, intensive care unit

### Study quality

The mean Newcastle-Ottawa quality score was 8.5 (range 4–9). The majority of full text studies were high quality (*n* = 36, 90%), while a few were moderate quality (*n* = 4, 10%); with no observational studies rated as poor quality. Of the remaining studies identified (*n* = 11, 21.6%), quality assessment was not possible due to insufficient data as studies were only available in abstract form.

### Indicators of capacity strain

A total of 16 potential strain indicators were identified 110 times in our included citations, and classified as structure (*n* = 4, 25%), process (*n* = 7, 44%) and outcome (*n* = 5, 31%) indicators, respectively (Table 2). The most commonly identified strain indicators were ICU acuity (*n* = 21; 19.1% [process]), ICU readmission (*n* = 18; 16.4% [outcome]), after-hours discharge (*n* = 15; 13.6% [process]) and ICU census (*n* = 13; 11.8% [structure]). There was substantial heterogeneity in the operational definitions used to define strain indicators across studies (Table 2). Strain indicators were also stratified across the six dimension of healthcare quality: safety (*n* = 4; 25%); effectiveness (*n* = 3; 19%); patient-centeredness (*n* = 1; 6%); timeliness (*n* = 3; 19%); efficiency (*n* = 4; 25%); and equitability (*n* = 1; 6%) [18].

**Table 2:** Summary of description and definitions for strain indicators across studies

Quality indicator	description	Definitions used in the literature for exposure, outcome and analysis
ICU census	ICU bed occupancy ICU bed availability	• Total number of patients who spent at least 2 h in the ICU on the calendar day the patient was admitted. • Bed occupancy. • ICU full and not able to admit or discharge any patients. • No available ICU beds
Queuing	Time delay in patient ICU admission	• Delay in ICU admission. • Delay in ICU admission > 4 h. • Delay in ICU admission > 6 h. • Delay in ICU admission > 8 h. • Mean time from bed request to ICU transfer.
Nurse-to-patient ratio	Ratio of nurses to patient for a given ICU.	• Ratio of nurses to beds in an ICU. • Ratio > or < 1:2. • Ratio 1:2 vs. 1:2.
Daily rounds by intensivist	Daily review of patient’s condition and problem list by MRP.	• No definition provided.
ICU transfer	Transfer of an ICU patient from one ICU to another.	• Inter-hospital transfer of an ICU patient.
Acuity	Severity of illness of patients in the ICU.	• APACHE II score. • Acute physiology score. • MPM-0 score.
After-hours discharges	ICU discharge of a patient to the hospital ward outside of regular hours.	• ICU discharge between 1600 and 0800 h. • ICU discharge between 1800 and 0600 h. • ICU discharge between 2000 and 0800 h. • ICU discharge between 2200 and 0700 h.
Turnover	The number of new admissions to and discharges from an ICU over a given time period.	• Number of new admissions, discharges and transfers. • Number of new admissions per day. • Number of admissions in a given week.
Workload	Intensity of bedside nurse work required per patient per unit of time.	• Number of new patient admissions and number of patient-care days. • Volume and pressure of work. • TISS score
Early ICU discharge	Premature ICU discharge.	• Discharged early but would have benefited from longer ICU stay.
Refusal rate	A measure of the number of patients referred to but not admitted to the ICU.	• Patients who were referred to but not admitted to the ICU.
ICU readmission	Patients who have been discharged from the ICU and are readmitted within the same hospitalization.	• ICU readmission within 24 h. • ICU readmission within 48 h. • ICU readmission within 72 h. • Unplanned ICU readmission.
SMR	Ratio between the observed number of deaths in a study population and the number of deaths that would be expected, based on age and sex-specific rates or severity of illness score.	• Not applicable.
Burnout	Workplace-related psychological stress leading to healthcare providers perception of emotional exhaustion, depersonalization and lack of personal achievement.	• State characterized by physical and/or psychological fatigue, disappointment, underachievement, tiredness and desire to leave work.
Job satisfaction	Healthcare workers satisfaction with work and workplace environment.	• Nursing self-reports of either being satisfied, unsatisfied or partially satisfied.
Surgery cancellation	Elective surgeries that is postponed or cancelled due to ICU bed availability.	• Cancellation of surgery due to lack of ICU bed. • Surgery cancelled or rescheduled.

This table shows examples of varying definitions across the retrieved studies of the most common ‘same’ strain indicators

*Abbreviations*: *APACHE* Acute Physiology and Chronic Health Evaluation, *ICU* intensive care unit, *MPM* mortality prediction model, *MRP* most responsible physician, *SMR* standardized mortality ratio, *TISS* Therapeutic Intervention Scoring System,

Above are shown description of the context and specific definitions of the most common 'same' strain indicators from included studies. Selected strain measures (i.e., daily rounds by an intensivist; SMR) were not precisely defined and assumed

### National quality measurement and reporting criteria

Features of these potential strain indicators, as discussed by study authors, generally focused on importance as key ICU performance or quality indicators (*n* = 95; 74%), followed by scientific acceptability (*n* = 23; 24%) and then by usability and feasibility (*n* = 11; 12%) (Table 3). Importance was further stratified across specific elements of capacity strain, including importance to patient-related outcomes (*n* = 47; 55%), importance as an indicator of ICU operations and organizational planning (*n* = 42; 44%), and importance to healthcare costs (*n* = 6; 6%). None of the studies presented data regarding the reliability or validity of the performance of the indicators.

**Table 3:** Categorization and relevance of identified quality indicators

Categorization of strain measure by the Donabedian framework^†^	Importance^¶^ (*n* = 95)			Scientific acceptability^¶^ (*n* = 23)	Usability and feasibility^¶^ (*n* = 11)	
	Quality (*n* = 42)	Patient-centered outcomes (*n* = 47)	Healthcare costs (*n* = 6)		Operational (*n* = 8)	Integrate into EHR (*n* = 3)
Structure (*n* = 30)						
1. ICU census (*n* = 13)	7	7	1	4	3	1
2. Queuing (*n* = 11)	2	3	1	3	1	–
3. Nurse to patient ratio (*n* = 5)	2	3	1	2	1	–
4. Daily rounds by intensivist (*n* = 1)	1	1	–	–	–	–
Process (*n* = 50)						
5. ICU transfer (*n* = 1)	–	–	–	–	–	–
6. ICU acuity (*n* = 21)	10	10	–	2	–	–
7. After-hours discharge (*n* = 15)	11	11	2	8	1	–
8. Turnover (*n* = 4)	2	2	–	–	–	–
9. Workload (*n* = 4)	1	1	–	–	1	1
10. Early ICU discharge (*n* = 3)	–	–	–	–	–	–
11. Refusal rate (*n* = 2)	–	–	–	–	–	–
Outcome (*n* = 30)						
12. ICU readmission (*n* = 18)	7	7	1	2	–	–
13. SMR (*n* = 4)	1	1	–	–	1	1
14. Burnout (*n* = 2)	–	–	–	1	–	–
15. Job satisfaction (*n* = 2)	–	–	–	1	–	–
16. Surgery cancellation (*n* = 2)	–	–	–	–	–	–

In the first column, the types of identified QIs are listed with the number of instances in parenthesis. In the subsequent columns the breakdown of the characteristics of the identified QIs as per the four criteria proposed by the United States Strategic Framework Board for a National Quality Measurement and Reporting System. Importantly, not all QIs had these characteristics described in the identified studies

*Abbreviations*: *EHR* electronic health record, *ICU* intensive care unit, *SMR* standardized mortality ratio

^†^Strain measures are stratified by structure, process, or outcome

^¶^The number of instances that each quality indicator was deemed relevant as per the authors according to the US National quality measurement and reporting criteria are also listed

## Discussion

Strain on ICU capacity is complex; however, strain has recognized implications for the practice of critical care, consistently showing association with altered care processes, suboptimal care delivery, adverse patient outcomes, and a negative workplace environment [1]. In response to not being able to identify a prior appraisal characterizing indicators of strained ICU capacity, we performed a rigorous systematic review and evidence synthesis.

### Summary of key findings

First, we identified 16 potential indicators of strained ICU capacity that encompassed all three domains of the Donabedian framework and quality dimensions. Second, we found that the operational definitions for strain indicators across studies were substantially heterogeneous. For example, we found 110 examples of indicators in our retrieved studies; and due to varied definitions, these were subsequently consolidated into related themes. Third, few studies had evaluated the scientific acceptability, usability or feasibility of the proposed strain indicators. Similarly, none of the studies identified specifically described the reliability or validity of the performance of these potential indicators of strain. Fourth, the most common strain indicators identified included indicators of ICU acuity, ICU readmission, after-hours discharge, and ICU census/occupancy. Notably, these strain indicators have considerable overlap with commonly recommended key indicators of ICU performance (KPI) [19, 20]. Finally, we also identified several indicators that while perhaps also analogous with some ICU KPIs, may also be suitable to characterize strained capacity conditions. These specific structure (e.g., queuing; nurse-to-patient ratios), process (e.g., bed turnover; workload; refusal rate) and outcome (e.g., healthcare professional burnout; surgery postponements) indicators could be evaluated over both the short-term and intermediate-term to provide holistic data on contributors to and effects of strained ICU capacity.

### Context with prior literature

Population growth, advances in medical science and improved capability to support critically ill patients have all translated into a sustained and rising demand for critical care services [21]. This increased demand; however, cannot be universally accompanied by an increased supply of ICU resources, which are costly [22]. The costs of expanding ICU extend beyond the “ICU bed” per se and necessitate considerable investment in human resources, specialized equipment (i.e., mechanical ventilators), and supplies to sustainably operate. Moreover, the supply side of ICU services is not standardized and are highly inconsistent across jurisdictions for reasons that are not based on evidence-informed scientific assessment of need [23, 24]. Regardless of the reasons, mismatches between demand and supply for ICU services are increasingly encountered [7]. This mismatch on any given day in any given ICU will create strain on that ICU’s capacity to accommodate the next critically ill patient. Strained ICUs are at risk of providing suboptimal quality of care, and risk higher rates of adverse events, premature discharges, unplanned readmissions and death [4, 25–28]. Arguably, sustained strain contributes to inefficient and inequitable utilization of finite ICU resources. While opening additional ICU beds may seem the simplest response, this is not necessarily sustainable, and likely only a short-term reprieve [29]. Rather, in order to explain the breadth of effect strain may have on patients, professionals and operations, a constellation of evidence-informed quality indicators are likely to be necessary.

Many of the strain indicators identified in our review are highly correlated with commonly cited ICU KPI or quality indicators [19, 20]. However, we contend many of these indicators have not been specifically evaluated in the context of strained operating conditions and capacity. Moreover, many will exhibit context-specific variation and require local or regional evaluation. Commonly described indicators of strain capacity, particularly occupancy/bed availability, acuity, and numbers of new admissions have repeatedly shown association with adverse outcomes, deviations in care processes, and changes in resource use [3, 5–7, 9, 10, 30]. However, it remains uncertain whether these alone may completely capture the spectrum of effects for how strain may manifest and exert its effects across heterogeneous ICU settings. Rather, they may put focus on queuing rather than on demand and the capacity to manage high flow and increased patient turnover, such as in specialized ICUs (i.e., cardiac surgery) [31]. Complementary indicators of strain may be important in such circumstances.

ICU readmission has been recognized as a potential indicator of strained ICU capacity [10]. While ICU readmission has been endorsed as a standard KPI for ICUs [19, 20]; there is uncertainty to its validity [32]. In a retrospective cohort study, Wagner et al. found greater strain, defined by average census, number of new ICU admissions, and ICU acuity, was associated with a shorter ICU stay and a small but significant increased risk for ICU readmission following discharge [10]. However, among those readmitted, there is uncertainty whether these events were avoidable or attributable to premature triage in response to strained conditions. Recent observations suggest the majority of ICU readmissions are not preventable or attributable to causal actions or omissions of the ICU team [33]. Further evaluation of ICU readmission as a potential indicator of strain should aim to integrate additional indicators of strain and adjudication of whether ICU readmission was avoidable.

### Limitations

While our rigorous review identified 51 studies that characterized a spectrum of indicators of strained capacity across a range of quality dimensions, some of which are likely routinely measured or should be implemented by ICUs, our findings should be considered in the context of the following limitations. The identified indicators across studies were variably defined, were often not the intended primary exposure, and the operational characteristics were incomplete described (e.g., elements identified in the US Strategic Framework Board for a National Quality Measurement and Reporting System) (Table 3). Further refinement and streamlining of definitions will be needed prior to operationalizing these potential identified strain indicators. Furthermore, as for the abovementioned reasons, pooled analyses across “strain indicators” were not feasible.

### Implications for healthcare professionals, health policy and research

The challenge for healthcare professionals is to clearly understand when and to what extent strain is negatively impacting their decision-making, the quality of care provided and the performance of a given ICU, and to readily identify and respond to factors most responsible. Healthcare professionals should be particularly mindful of the influence strained ICU capacity may have to modify behaviours and care processes [5–7]. There is uncertainty on how best to measure strained capacity. While simple objective indicators such as ICU census/occupancy are strongly perceived to indicate strain [34], the spectrum of how strain can manifest is likely far most complex and it is unlikely that any single indicator will satisfy all the potential domains from which strain may originate. Moreover, as a consequence, there may be limited appreciation for the collateral effect of strained capacity, such as a negative impact on ambulance offloads, emergency department crowding, and postponements of elective surgery.

Simple, translatable and easily reportable indicators are needed to identify and quantify strained capacity. Widespread implementation of electronic health records (EHR) and data repositories/registries have enabled the routine calculation and reporting of standard ICU KPIs [3]. Such infrastructure could be re-orientated to generate a concise dashboard or index consisting of strain indicators across multiple quality domains (Table 4). For example, 13 of our proposed indicators could readily be integrated into a strain dashboard. Additional potential indicators, including healthcare professional burnout (i.e., attribution, absenteeism, overtime), workplace satisfaction, patient-family satisfaction would require additional resources to integrate. We contend this is a fundamental step towards developing innovative quality improvement interventions aimed to improve safety (i.e., care processes, adverse events, nosocomial infections), effectiveness (i.e., SMR), timeliness (e.g., access, queuing), efficiency (e.g., flow, avoidable ICU days), and to better equip ICUs to anticipate and manage strained capacity. Future work should aim to evaluate the feasibility of implementing a constellation of strain indicators, evaluate the association of strain indicators on patient-centered and health resource outcomes, including impact on additional KPIs, establish threshold for optional strain indicator definitions and benchmarks (Table 5), recognizing some may be geographically and context-specific. Finally, future work should consider the development of indicators that try to capture the match between the demand and capacity, with the goals of having ICUs operate in an ideal range to limit both strain and healthcare inefficiencies.

**Table 4:** Proposed dashboard of indicators for ICU strain

Short-term measures (e.g., daily or weekly)	Intermediate term measures (e.g., monthly or quarterly)
ICU acuity	ICU readmission
After-hours discharges	Burnout
ICU census	Workplace satisfaction
Sedation interruption*	Early ICU discharge
Queuing	Surgery cancellation
Mobilization*	ICU transfer
Nurse-to-patient ratio	Refusal rate
Turnover	Adverse events*
Mechanical ventilation weaning*	SMR
Workload	Family satisfaction*
Daily rounds by intensivist	

Above are listed both short-term (i.e., measured daily) and intermediate-term (i.e., measured monthly or quarterly) QIs for ICU strain

*Abbreviations*: *ICU* intensive care unit*SMR* standardized mortality rate

^*^Proposed QIs that were not identified in our search strategy

**Table 5:** Proposed definitions and benchmarks for indicators of ICU strain

Quality Indicator	Proposed aggregate definition	Justification	Proposed benchmark or measure
ICU census	ICU not able to admit any new patients.	The exact percentage bed occupancy is less important than having capacity to admit.	< 10% of time
Queuing	Delay in time from orders to admit to ICU to ICU arrival.	Increasing delays for ICU admission result in suboptimal care for these critically ill patients. The most common timeframe in the literature was within 4 h of decision to admit.	< 4 h
Nurse-to-patient ratio	The number of nurses caring per patient.	A lower ratio of nurses per patient means less time can be spent per individual patient and increases nursing workload. To most common ratio studied in the literature was 1:2.	Adjusted nursing workload of < 1:2
Daily rounds by intensivist	Daily bedside visit by MRP to review patients’ medical condition and problem list.	Daily in-person rounds are critical when caring for ICU patients. These should occur daily in a formal fashion.	100%
ICU transfer	Inter-hospital transfer of an ICU patient due to lack of capacity.	This definition interplays with that of ICU census; however, it is an extension of the above, indicating that there are no mechanisms for increasing capacity at the strained institution.	None
ICU acuity	The average severity of illness of patients in the ICU.	More acutely ill patients provide both a physical and mental strain on ICU staff. The APACHE II score was most commonly used in the literature. However, institutionally specific scores may be used as well.	APACHE II Score
After-hours discharges	Unplanned discharges from the ICU outside of regular hours (as defined in per each individual institution)	Patients discharged outside of regular hours may not be evaluated by medical staff in a timely fashion. There were many definitions of ‘after-hours’ in the medical literature. ‘After-hours’ should relate to individual institutional practices.	None
Turnover	The number of admissions and discharges from an ICU per 24-h period.	Typically highest patient workload occurs on ICU admission and discharge.	n/a
Workload	The volume and pressure of work.	Higher workload can lead to increased stress and concerns regarding patient safety. An objective measure of workload is necessary to quantify this variable.	TISS-28 Score
Early ICU Discharge	Discharge from an ICU earlier than preferable as per the MRP.	Physicians must triage patients at time of ICU capacity strain to ensure that the sickest patients be those located in the ICU. This may require immediate decision-making regarding discharging of less acutely ill patients.	None
Refusal Rate	The ratio of patients refused entry to the ICU vs. total number of ICU consults.	As strain in the ICU increases, physicians are less likely to admit patients who may not truly require ICU level care. This needs to be balanced with referred patients who do not require ICU level care.	0% of appropriate ICU consultations
ICU Readmission	Avoidable ICU readmission within 48 h of discharge as adjudicated by admitting physician.	Most ICU readmissions are unavoidable and hence are not a reflection of ICU strain or quality. However, if an ICU is under strain and patients are discharged prematurely and this results in ICU readmissions, this may be a marker of strain. Avoidable readmissions should be adjudicated as per the admitting physician.	None
Standardized mortality ratio	Ratio between the observed number of deaths in a study population and the number of deaths that would be expected, based on age and sex-specific rates in a standard population and the age and sex distribution of the study population.	An increasing varying SMR may be related to varying ICU strain. Benchmark is based on data from all Alberta provincial ICUs.	< 15%
Burnout	Work-related stress leading to feelings of pressured, overwhelmed and desire to leave work.	As workload and patient acuity increases, healthcare providers may themselves feel overwhelmed and unable to carry on work. An objective measure of burnout syndrome (BoS) is necessary to quantify this variable and the Maslach Burnout Inventory has been extensively studied in the literature and may be referenced across ICUs.	Maslach Burnout Inventory
Job satisfaction	Healthcare workers reporting lack of satisfaction with their job.	With increasing strain and stress at the workplace, there is decreasing satisfaction on the job. An objective measure is necessary to quantify this variable.	Measurement of Job Satisfaction
Surgery cancellation	Surgeries that require cancelation of rescheduling due to ICU constrains.	Certain elective surgeries necessitate post-operative ICU monitoring. However, in cases of strain, these surgeries may be cancelled or rebooked.	None

A proposed aggregate definition for each quality indicator is given above. Where applicable a benchmark for these indicators, along with rationale for its selection is given. When not applicable, an indicator quantifying these quality indicators is proposed so as to stratify amongst different ICUs.

*Abbreviations*: *APACHE* Acute Physiology and Chronic Health Evaluation, *ICU* intensive care unit, *MRP* most responsible physician, *SMR* standardized mortality ratio

## Conclusions

Strained ICU capacity is likely to increasingly be encountered concomitant with growing demand for critical care and ICU resources. Strain negatively impacts a wide variety of care processes and outcomes for patients, families, healthcare professionals and the healthcare system. Identification of strained capacity is complex and ideally requires an evidence-informed set of indicators. We have characterized 16 indicators of strained ICU capacity across the spectrum of healthcare quality domains. Future work should focus on further rigorously defining indicated of strained capacity, on evaluating the acceptability and feasibility of implementation, and on assessing their value for identifying strain and evaluating interventions to manage strained ICU capacity.

## Key messages

- Strained ICU capacity is associated with alterations in care processes and adverse outcomes.
- This systematic review has identified and characterized 16 potential indicators of strained ICU capacity.
- Indicators were variable in their operational definitions and few were evaluated for scientific acceptability, usability or feasibility.
- The most common indicators of strain showed overlap with recommended ICU key performance indicators (i.e., ICU acuity, ICU readmission, after-hours discharge, and occupancy).
- Several indicators of strain could readily be implemented and would likely add value, particularly if clustered as a dashboard or index, to provide holistic ICU-specific information on key contributors to strain.

## Additional files

Additional file 1: PRISMA-P checklist. (DOC 62 kb)

Additional file 2: Search strategy. (DOCX 22 kb)

Additional file 3: Grey literature sources. (DOCX 45 kb)

Additional file 4: References of included studies. (DOCX 156 kb)

## Abbreviations

ARCHE: Alberta Research Centre for Health Evidence
CENTRAL: Cochrane Central Register of Controlled Trials
HER: Electronic health record
ICU: intensive care unit
KPI: Key performance indicator
NOS: Newcastle-Ottawa Quality Assessment Scale
SMR: Standardized mortality ratio
