# Supplementary material for: Indicators of intensive care unit capacity strain: a systematic review
Source: Crit Care. 2018 Mar 27;22:86. doi: 10.1186/s13054-018-1975-3 (PMC5870068; doi:10.1186/s13054-018-1975-3)
Supplement: Supplementary file 2 — Search strategy. (DOCX 22 kb) [file 13054_2018_1975_MOESM2_ESM.docx]

**Additional File 2: Search Strategy**

**Database**: Ovid MEDLINE(R) In-Process & Other Non-Indexed Citations and Ovid MEDLINE(R) 1946 to Present

**Original search conducted**: 11 August 2015

**Update search conducted**: 1 February 2016

**Strategy**:

1. exp Critical Care/

2. exp Critical Care Nursing/

3. Critical Illness/

4. Intensive Care Units/

5. exp Intensive Care Units, Pediatric/

6. Multiple Organ Failure/

7. critical care.tw,kf.

8. (critical* adj2 (department* or unit*)).tw,kf.

9. critical* ill*.tw,kf.

10. intensive care*.tw,kf.

11. (intensive adj2 (department* or unit*)).tw,kf.

12. intensivist*.tw,kf.

13. (ICU* or NICU* or PICU*).tw,kf.

14. (multi* organ adj (disfunction* or dis function* or dysfunction* or dys function* or failure*)).tw,kf.

15. (multi* system adj (disfunction* or dis function* or dysfunction* or dys function* or failure*)).tw,kf.

16. or/1-15 [Combined MeSH terms and keywords for critical care]

17. Delivery of Health Care/

18. Efficiency/

19. Efficiency, Organizational/

20. Health Services Research/

21. exp Medical Audit/ and (ec or og or sn or ut).fs.

22. "Outcome Assessment (Health Care)"/

23. patient outcome assessment/

24. exp Program Evaluation/

25. Quality Assurance, Health Care/

26. Quality Improvement/

27. Quality Indicators, Health Care/

28. Quality of Health Care/

29. (bench mark* or benchmark*).tw,kf.

30. (delivery adj2 health*).tw,kf.

31. (efficien* adj2 (assess* or assurance* or evaluat* or improv* or indicat* or measur* or test*)).tw,kf.

32. (health* adj2 service* research).tw,kf.

33. (performance adj2 (assess* or assurance* or evaluat* or improv* or indicat* or measur* or test*)).tw,kf.

34. (outcome* adj2 (assess* or assurance* or evaluat* or improv* or indicat* or measur* or test*)).tw,kf.

35. (program* adj2 (assess* or assurance* or evaluat* or improv* or indicat* or measur* or test*)).tw,kf.

36. or/17-35 [Combined MeSH terms and keywords for quality]

37. Absenteeism/

38. Bed Occupancy/

39. Burnout, Professional/

40. exp Hospital Bed Capacity/

41. exp Health Services Accessibility/

42. exp Job Satisfaction/

43. exp Patient Discharge/

44. exp Patient Readmission/

45. exp Resource Allocation/

46. Waiting Lists/

47. absenteeism*.tw,kf.

48. (access* adj2 service*).tw,kf.

49. ((admiss* or admit*) adj2 (deni* or deny or refus*)).tw,kf.

50. ((allocat* or distribut*) adj2 resource*).tw,kf.

51. ((bed or beds) adj3 (capacit* or occupan*)).tw,kf.

52. (burn* out or burnout).tw,kf.

53. (capacity adj2 (limit* or strain*)).tw,kf.

54. ((job or jobs or profession*) adj2 satisf*).tw,kf.

55. ((pre mature* or premature*) adj discharg*).tw,kf.

56. queu*.tw,kf.

57. rationing*.tw,kf.

58. (re admission* or readmission*).tw,kf.

59. wait* list*.tw,kf.

60. or/37-59 [Combined MeSH terms and keywords for capacity strain]

61. and/16,36,60 [Combination of ICU, quality and capacity strain terms]

62. exp animals/ not humans.sh.

63. 61 not 62 [human filter]

64. limit 63 to yr="1990-current" [publication date limit]

65. limit 64 to english [language limit]

66. remove duplicates from 65 (1655)

**Database**: Ovid Embase 1988 to 2015 Week 32

**Original search conducted**: 13 August 2015

**Update search conducted**: 1 February 2016

**Strategy**:

1. critical illness/

2. intensive care/

3. exp intensive care nursing/

4. intensive care unit/

5. multiple organ failure/

6. newborn intensive care/

7. critical care.tw.

8. (critical* adj2 (department* or unit*)).tw.

9. critical* ill*.tw.

10. intensive care*.tw.

11. (intensive adj2 (department* or unit*)).tw.

12. intensivist*.tw.

13. (ICU* or NICU* or PICU*).tw.

14. (multi* organ adj (disfunction* or dis function* or dysfunction* or dys function* or failure*)).tw.

15. (multi* system adj (disfunction* or dis function* or dysfunction* or dys function* or failure*)).tw.

16. or/1-15 [Combined Emtree terms and keywords for critical care]

17. health care quality/

18. health services research/

19. medical audit/

20. organizational efficiency/

21. productivity/

22. exp program evaluation/

23. quality control/

24. total quality management/

25. (bench mark* or benchmark*).tw.

26. (delivery adj2 health*).tw.

27. (efficien* adj2 (assess* or assurance* or evaluat* or improv* or indicat* or measur* or test*)).tw.

28. (health* adj2 service* research).tw.

29. (performance adj2 (assess* or assurance* or evaluat* or improv* or indicat* or measur* or test*)).tw.

30. (program* adj2 (assess* or assurance* or evaluat* or improv* or indicat* or measur* or test*)).tw.

31. or/17-30 [Combined Emtree terms and keywords for quality]

32. absenteeism/

33. burnout/

34. hospital bed capacity/

35. hospital bed utilization/

36. hospital discharge/

37. hospital readmission/

38. job satisfaction/

39. resource allocation/

40. absenteeism*.tw.

41. (access* adj2 service*).tw.

42. ((admiss* or admit*) adj2 (deni* or deny or refus*)).tw.

43. ((allocat* or distribut*) adj2 resource*).tw.

44. ((bed or beds) adj3 (capacit* or occupan*)).tw.

45. (burn* out or burnout).tw.

46. (capacity adj2 (limit* or strain*)).tw.

47. ((job or jobs or profession*) adj2 satisf*).tw.

48. ((pre mature* or premature*) adj discharg*).tw.

49. queu*.tw.

50. rationing*.tw.

51. (re admission* or readmission*).tw.

52. wait* list*.tw.

53. or/32-52 [Combined Emtree terms and keywords for capacity strain]

54. and/16,31,53 [Combination of ICU, quality and capacity strain terms]

55. animals/ not (animals/ and humans/)

56. 54 not 55 [human filter]

57. limit 56 to yr="1990-current" [publication date limit]

58. limit 57 to english [language limit] (1572)

**Database**: CINAHL Plus with Full Text via EBSCOhost

**Search conducted**: 14 August 2015

**Strategy**:

1. (MH "Critical Care")

2. (MH "Critical Care Nursing+")

3. (MH "Critical Illness")

4. (MH "Intensive Care, Neonatal")

5. (MH "Intensive Care Units")

6. (MH "Intensive Care Units, Pediatric+")

7. (MH "Multiple Organ Dysfunction Syndrome")

8. "critical care"

9. critical* N2 (department* or unit*)

10. "critical* ill*"

11. "intensive care*"

12. intensive N2 (department* or unit*)

13. intensivist*

14. ICU* or NICU* or PICU*

15. "multi* organ" N1 (disfunction* or "dis function*" or dysfunction* or "dys function*" or failure*)

16. "multi* system" N1 (disfunction* or "dis function*" or dysfunction* or "dys function*" or failure*)

17. S1 OR S2 OR S3 OR S4 OR S5 OR S6 OR S7 OR S8 OR S9 OR S10 OR S11 OR S12 OR S13 OR S14 OR S15 OR S16

18. (MH "Benchmarking")

19. (MH "Health Care Delivery")

20. (MH "Health Services Research+")

21. (MH "Nursing Audit")

22. (MH "Organizational Efficiency")

23. (MH "Outcome Assessment")

24. (MH "Process Assessment (Health Care)+")

25. (MH "Productivity")

26. (MH "Quality Assurance+")

27. (MH "Quality of Care Research")

28. (MH "Quality of Health Care")

29. (MH "Quality Improvement")

30. "bench mark*" or benchmark*

31. delivery N2 health*

32. efficien* N2 (assess* or assurance* or evaluat* or improv* or indicat* or measur* or test*)

33. health* N2 "service* research"

34. performance N2 (assess* or assurance* or evaluat* or improv* or indicat* or measur* or test*)

35. outcome* N2 (assess* or assurance* or evaluat* or improv* or indicat* or measur* or test*)

36. S18 OR S19 OR S20 OR S21 OR S22 OR S23 OR S24 OR S25 OR S26 OR S27 OR S28 OR S29 OR S30 OR S31 OR S32 OR S33 OR S34 OR S35

37. (MH "Absenteeism")

38. (MH "Bed Occupancy")

39. (MH "Health Services Accessibility+")

40. (MH "Job Satisfaction+")

41. (MH "Patient Discharge+")

42. (MH "Readmission")

43. (MH "Resource Allocation+")

44. (MH "Waiting Lists")

45. absenteeism*

46. access* N2 service*

47. (admiss* or admit*) N2 (deni* or deny or refus*)

48. (allocat* or distribut*) N2 resource*

49. (bed or beds) N3 (capacit* or occupan*)

50. "burn* out" or burnout

51. capacity N2 (limit* or strain*)

52. (job or jobs or profession*) N2 satisf*

53. ("pre mature*" or premature*) N1 discharg*

54. queu*

55. rationing*

56. "re admission*" or readmission*

57. "wait* list*"

58. S37 OR S38 OR S39 OR S40 OR S41 OR S42 OR S43 OR S44 OR S45 OR S46 OR S47 OR S48 OR S49 OR S50 OR S51 OR S52 OR S53 OR S54 OR S55 OR S56 OR S57

59. S17 AND S36 AND S58

60. (MH "Animals+") not (MH "Humans")

61. S59 NOT S60

62. S59 NOT S60 Limiters – Publication Date: 19900101-20151231 ; Language: English (971)

**Database**: Cochrane Library via Wiley

**Search conducted**: 14 August 2015

**Strategy**:

1. [mh "Critical Care"]

2. [mh "Critical Care Nursing"]

3. [mh ^"Critical Illness"]

4. [mh ^"Intensive Care Units"]

5. [mh "Intensive Care Units, Pediatric"]

6. [mh ^"Multiple Organ Failure"]

7. "critical care":ti,ab,kw

8. (critical* NEAR/2 (department* or unit*)):ti,ab,kw

9. "critical* ill*":ti,ab,kw

10. "intensive care*":ti,ab,kw

11. (intensive NEAR/2 (department* or unit*)):ti,ab,kw

12. intensivist*:ti,ab,kw

13. (ICU* or NICU* or PICU*):ti,ab,kw

14. ("multi* organ" NEXT (disfunction* or "dis function*" or dysfunction* or "dys function*" or failure*)):ti,ab,kw

15. ("multi* system" NEXT (disfunction* or "dis function*" or dysfunction* or "dys function*" or failure*)):ti,ab,kw

16. {OR #1-#15}

17. [mh ^"Delivery of Health Care"]

18. [mh ^Efficiency]

19. [mh ^"Efficiency, Organizational"]

20. [mh ^"Health Services Research"]

21. [mh "Medical Audit"]

22. [mh ^"Outcome Assessment (Health Care)"]

23. [mh ^"Patient Outcome Assessment"]

24. [mh "Program Evaluation"]

25. [mh ^"Quality Assurance, Health Care"]

26. [mh ^"Quality Improvement"]

27. [mh ^"Quality Indicators, Health Care"]

28. [mh ^"Quality of Health Care"]

29. ("bench mark*" or benchmark*):ti,ab,kw

30. (delivery NEAR/2 health*):ti,ab,kw

31. (efficien* NEAR/2 (assess* or assurance* or evaluat* or improv* or indicat* or measur* or test*)):ti,ab,kw

32. (health* NEAR/2 "service* research"):ti,ab,kw

33. (outcome* NEAR/2 (assess* or assurance* or evaluat* or improv* or indicat* or measur* or test*)):ti,ab,kw

34. (performance NEAR/2 (assess* or assurance* or evaluat* or improv* or indicat* or measur* or test*)):ti,ab,kw

35. (program* NEAR/2 (assess* or assurance* or evaluat* or improv* or indicat* or measur* or test*)):ti,ab,kw

36. {OR #17-#35}

37. [mh ^Absenteeism]

38. [mh ^"Bed Occupancy"]

39. [mh ^"Burnout, Professional"]

40. [mh "Hospital Bed Capacity"]

41. [mh "Health Services Accessibility"]

42. [mh "Job Satisfaction"]

43. [mh "Patient Discharge"]

44. [mh "Patient Readmission"]

45. [mh "Resource Allocation"]

46. [mh ^"Waiting Lists"]

47. absenteeism*:ti,ab,kw

48. (access* NEAR/2 service*):ti,ab,kw

49. ((admiss* or admit*) NEAR/2 (deni* or deny or refus*)):ti,ab,kw

50. ((allocat* or distribut*) NEAR/2 resource*):ti,ab,kw

51. ((bed or beds) NEAR/3 (capacit* or occupan*)):ti,ab,kw

52. ("burn* out" or burnout):ti,ab,kw

53. (capacity NEAR/2 (limit* or strain*)):ti,ab,kw

54. ((job or jobs or profession*) NEAR/2 satisf*):ti,ab,kw

55. (("pre mature*" or premature*) NEXT discharg*):ti,ab,kw

56. queu*:ti,ab,kw

57. rationing*:ti,ab,kw

58. ("re admission*" or readmission*):ti,ab,kw

59. "wait* list*":ti,ab,kw

60. {OR #37-#59}

61. {AND #16,#36,#60}

62. {AND #16,#36,#60} Publication Year from 1990 to 2015, English (120)

**Database**: Web of Science Core Collection (Includes: Science Citation Index Expanded (1900-present); Social Sciences Citation Index (1900-present))

**Search conducted**: 24 August 2015

**Strategy**:

1. TS="critical care"

2. TS=(critical NEAR/2 (department* OR unit*))

3. TS="critical* ill*"

4. TS="intensive care*"

5. TS=(intensive NEAR/2 (department* OR unit*))

6. TS=intensivist*

7. TS=(ICU* OR NICU* OR PICU*)

8. TS=("multi* organ" NEAR/1 (disfunction* OR "dis function*" OR dysfunction* OR "dys function*" OR failure*))

9. TS=("multi* system" NEAR/1 (disfunction* OR "dis function*" OR dysfunction* OR "dys function*" OR failure*))

10. #1 OR #2 OR #3 OR #4 OR #5 OR #6 OR #7 OR #8 OR #9 [Combined keywords for critical care]

11. TS=(delivery NEAR/2 health*)

12. TS=(efficien* NEAR/2 (assess* OR assurance* OR evaluat* OR improv* OR indicat* OR measur* OR test*))

13. TS=(health* NEAR/2 "service* research")

14. TS=(performance NEAR/2 (assess* OR assurance* OR evaluat* OR improv* OR indicat* OR measur* OR test*))

15. TS=(outcome* NEAR/2 (assess* OR assurance* OR evaluat* OR improv* OR indicat* OR measur* OR test*))

16. TS=(program* NEAR/2 (assess* OR assurance* OR evaluat* OR improv* OR indicat* OR measur* OR test*))

17. #11 OR #12 OR #13 OR #14 OR #15 OR #16 [Combined keywords for quality]

18. TS=absenteeism*

19. TS=(access* NEAR/2 service*)

20. TS=((admiss* OR admit*) NEAR/2 (deni* OR deny OR refus*))

21. TS=((allocat* OR distribut*) NEAR/2 resource*)

22. TS=((bed OR beds) NEAR/3 (capacit* OR occupan*))

23. TS=("burn* out" OR burnout)

24. TS=(capacity NEAR/2 (limit* OR strain*))

25. TS=((job OR jobs OR profession*) NEAR/2 satisf*)

26. TS=(("pre mature*" or premature*) NEAR/1 discharg*)

27. TS=queu*

28. TS=rationing*

29. TS=("re admission*" OR readmission*)

30. TS="wait* list*"

31. #18 OR #19 OR #20 OR #21 OR #22 OR #23 OR #24 OR #25 OR #26 OR #27 OR #28 OR #29 OR #30 [Combined keywords for capacity strain]

32. #10 AND #17 AND #31 [Combination of ICU, quality and capacity strain terms]

33. limit 32 to yr="1990-current" [publication date limit]

34. limit 33 to english [language limit] (576)
