# Supplementary material for: Indicators of intensive care unit capacity strain: a systematic review
Source: Crit Care. 2018 Mar 27;22:86. doi: 10.1186/s13054-018-1975-3 (PMC5870068; doi:10.1186/s13054-018-1975-3)
Supplement: Supplementary file 3 — Grey literature sources. (DOCX 45 kb) [file 13054_2018_1975_MOESM3_ESM.docx]

**Additional File 3: Grey Literature Sources**

1) Database of the AHRQ National Quality Measures Clearinghouse

2) Proceedings of the American Thoracic Society

3) Proceedings of the Australian and New Zealand Intensive Care Society

4) Proceedings of the Canadian Critical Care Society

5) Proceedings of the European Society of Intensive Care Medicine

6) Proceedings of the Society of Critical Care Medicine
