# Supplementary material for: Indicators of intensive care unit capacity strain: a systematic review
Source: Crit Care. 2018 Mar 27;22:86. doi: 10.1186/s13054-018-1975-3 (PMC5870068; doi:10.1186/s13054-018-1975-3)
Supplement: Supplementary file 4 — References of included studies. (DOCX 156 kb) [file 13054_2018_1975_MOESM4_ESM.docx]

**Additional File 4. References for Included Studies**

1. Ahmed M, Lumley G, Nourse S, Hurding A, Thomas A, Healy M, (2015) Out-of-hours discharge from critical care: Does it matter? Critical Care 19: S184

2. Ahrens LJO, Morrow BM, Argent AC, (2012) Prospective evaluation of patients referred for admission to a South African paediatric intensive care unit: Patient profiles, reason for admission or refusa. Southern African Journal of Critical Care 28 (1): 28-29

3. Al-Jaghbeer M, Tekwani S, Kahn J, Gunn S, (2015) Incidence and etiology of potentially preventable ICU readmissions. Critical Care Medicine 1): 195-196

4. Al-Khalid M, (2014) Customer satisfaction in intensive care unit. Journal of the Intensive Care Society 1): S30

5. Amaravadi RK, Dimick JB, Pronovost PJ, Lipsett PA, (2000) ICU nurse-to-patient ratio is associated with complications and resource use after esophagectomy. Intensive Care Medicine 26: 1857-1862

6. Aytekin A, Yilmaz F, Kuguoglu S, (2013) Burnout levels in neonatal intensive care nurses and its effects on their quality of life. Aust J Adv Nurs 31: 39-47

7. Society of Critical Care Medicine (2015) Critical Care Congress 2015. In: Editor (ed)^(eds) Book Critical Care Congress 2015. City, pp.

8. Barker RC, Flint NJ, (2010) Consequences of time of discharge from intensive care on mortality and readmission rates in a uk university teaching hospital. Intensive Care Medicine 36: S164

9. Beck DH, McQuillan P, Smith GB, (2002) Waiting for the break of dawn? The effects of discharge time, discharge TISS scores and discharge facility on hospital mortality after intensive care. Intensive Care Medicine 28: 1287-1293

10. Biuk S, Thomas J, (2015) Adverse outcome for patients discharged from the ICU between 22:00 and 06:59 hours. Critical Care Medicine 1): 237

11. Brown SE, Ratcliffe SJ, Halpern SD, (2014) An empirical comparison of key statistical attributes among potential ICU quality indicators. Critical Care Medicine 42: 1821-1831

12. Brown SE, Ratcliffe SJ, Halpern SD, (2013) An empirical derivation of the optimal time interval for defining ICU readmissions. Med Care 51: 706-714

13. Chalfin DB, Trzeciak S, Likourezos A, Baumann BM, Dellinger RP, (2007) Impact of delayed transfer of critically ill patients from the emergency department to the intensive care unit. Critical Care Medicine 35: 1477-1483

14. Cooper GS, Sirio CA, Rotondi AJ, Shepardson LB, Rosenthal GE, (1999) Are readmissions to the intensive care unit a useful measure of hospital performance? Med Care 37: 399-408

15. Czaja AS, Hosokawa PW, Henderson WG, (2013) Unscheduled readmissions to the PICU: epidemiology, risk factors, and variation among centers. Pediatric Critical Care Medicine 14: 571-579

16. Dara SI, Afessa B, (2005) Intensivist-to-bed ratio: association with outcomes in the medical ICU. Chest 128: 567-572

17. Duke GJ, Green JV, Briedis JH, (2004) Night-shift discharge from intensive care unit increases the mortality-risk of ICU survivors. Anaesth Intensive Care 32: 697-701

18. Duke GJ, Buist MD, Pilcher D, Scheinkestel CD, Santamaria JD, Gutteridge GA, Cranswick PJ, Ernest D, French C, Botha JA, (2009) Interventions to circumvent intensive care access block: a retrospective 2-year study across metropolitan Melbourne. Med J Aust 190: 375-378

19. Duke GJ, (2004) Metropolitan audit of appropriate referrals refused admission to intensive care. Anaesth Intensive Care 32: 702-706

20. Frankel HL, Foley A, Norway C, Kaplan L, (2006) Amelioration of increased intensive care unit service readmission rate after implementation of work-hour restrictions. J Trauma 61: 116-121

21. Frisho-Lima P, Gurman G, Schapira A, Porath A, (1994) Rationing critical care -- what happens to patients who are not admitted? Theor 9: 208-211

22. Gajic O, Malinchoc M, Comfere TB, Harris MR, Achouiti A, Yilmaz M, Schultz MJ, Hubmayr RD, Afessa B, Farmer JC, (2008) The Stability and Workload Index for Transfer score predicts unplanned intensive care unit patient readmission: initial development and validation. Critical Care Medicine 36: 676-682

23. Gantner D, Farley K, Bailey M, Huckson S, Hicks P, Pilcher D, (2014) Mortality related to after-hours discharge from intensive care in Australia and New Zealand, 2005-2012. Intensive Care Medicine 40: 1528-1535

24. Goldfrad C, Rowan K, (2000) Consequences of discharges from intensive care at night. Lancet 355: 1138-1142

25. Gopal S, Terry L, Corbett C, (2010) Association between out of hours discharge from the ICU and subsequent readmission. Critical Care 14: S159-S160

26. Harris SK, Rowan K, Singer M, Sanderson C, (2014) Delay to admission to critical care and mortality among deteriorating ward patients in 49 UK hospitals-results from (SPOT)light: A multi-site, prospective, observational cohort study. Intensive Care Medicine 1): S127

27. Hung SC, Kung CT, Hung CW, Liu BM, Liu JW, Chew G, Chuang HY, Lee WH, Lee TC, (2014) Determining delayed admission to the intensive care unit for mechanically ventilated patients in the emergency department. Critical Care 18

28. Iwashyna TJ, Kramer AA, Kahn JM, (2009) Intensive care unit occupancy and patient outcomes. Critical Care Medicine 37: 1545-1557

29. Joynt GM, Gomersall CD, Tan P, Lee A, Cheng CA, Wong EL, (2001) Prospective evaluation of patients refused admission to an intensive care unit: triage, futility and outcome. Intensive Care Medicine 27: 1459-1465

30. Kramer AA, Higgins TL, Zimmerman JE, (2013) The association between ICU readmission rate and patient outcomes. Critical Care Medicine 41: 24-33

31. Laupland KB, Misset B, Souweine B, Tabah A, Azoulay E, Goldgran-Toledano D, Dumenil AS, Vesin A, Jamali S, Kallel H, Clec'h C, Darmon M, Schwebel C, Timsit JF, (2011) Mortality associated with timing of admission to and discharge from ICU: a retrospective cohort study. BMC Health Services Research 11: 321

32. Leary T, Ridley S, Burchett K, Kong A, Chrispin P, Wright M, (2002) Assessing critical care unit performance: a global measure using graphical analysis. Anaesthesia 57: 751-755

33. Lim SC, Fok AC, Ong YY, (1996) Patient outcome and intensive care resource allocation using APACHE II. Singapore Med J 37: 488-491

34. Liu V, Kipnis P, Rizk NW, Escobar GJ, (2012) Adverse outcomes associated with delayed intensive care unit transfers in an integrated healthcare system. Journal of Hospital Medicine 7: 224-230

35. Louriz M, Abidi K, Akkaoui M, Madani N, Chater K, Belayachi J, Dendane T, Zeggwagh AA, Abouqal R, (2012) Determinants and outcomes associated with decisions to deny or to delay intensive care unit admission in Morocco. Intensive Care Medicine 38: 830-837

36. Nathanson BH, Higgins TL, Teres D, Copes WS, Kramer A, Stark M, (2007) A revised method to assess intensive care unit clinical performance and resource utilization. Critical Care Medicine 35: 1853-1862

37. European Society of Intensive Care Medicine (2014) ESICM Lives 2014. In: Editor (ed)^(eds) Book ESICM Lives 2014. City, pp.

38. Priestap FA, Martin CM, (2006) Impact of intensive care unit discharge time on patient outcome. Critical Care Medicine 34: 2946-2951

39. Pronovost PJ, Jenckes MW, Dorman T, Garrett E, Breslow MJ, Rosenfeld BA, Lipsett PA, Bass E, (1999) Organizational characteristics of intensive care units related to outcomes of abdominal aortic surgery. Jama 281: 1310-1317

40. Peterson JF, (2003) Accepting critically ill patients in transfer: how will it affect common hospital benchmarks? Journal of Clinical Outcomes Management 10: 464-465

41. European Society of Intensive Care Medicine (2012) ESICM Lives 2012. In: Editor (ed)^(eds) Book ESICM Lives 2012. City, pp.

42. Santamaria JD, Duke GJ, Pilcher DV, Cooper DJ, Moran J, Bellomo R, (2015) The timing of discharge from the intensive care unit and subsequent mortality. A prospective, multicenter study. American Journal of Respiratory & Critical Care Medicine 191: 1033-1039

43. Singh MY, Nayyar V, Clark PT, Kim C, (2010) Does after-hours discharge of ICU patients influence outcome? Crit Care Resusc 12: 156-161

44. Tobin AE, Santamaria JD, (2006) After-hours discharges from intensive care are associated with increased mortality. Med J Aust 184: 334-337

45. Town JA, Churpek MM, Yuen TC, Huber MT, Kress JP, Edelson DP, (2014) Relationship between ICU bed availability, ICU readmission, and cardiac arrest in the general wards. Critical Care Medicine 42: 2037-2041

46. Tucker J, Group UKNSS, (2002) Patient volume, staffing, and workload in relation to risk-adjusted outcomes in a random stratified sample of UK neonatal intensive care units: a prospective evaluation. Lancet 359: 99-107

47. Wagner J, Gabler NB, Ratcliffe SJ, Brown SE, Strom BL, Halpern SD, (2013) Outcomes among patients discharged from busy intensive care units. Ann Intern Med 159: 447-455

48. West E, Barron DN, Harrison D, Rafferty AM, Rowan K, Sanderson C, (2014) Nurse staffing, medical staffing and mortality in Intensive Care: An observational study. International Journal of Nursing Studies 51: 781-794

49. Amarasingham R, Swanson TS, Treichler DB, Amarasingham SN, Reed WG, (2010) A rapid admission protocol to reduce emergency department boarding times. Quality & Safety in Health Care 19: 200-204

50. Barado J, Esparza L, Cristina A, Mallor F, (2013) Analysis of the impact of medical decision-making on ICU bed management using a simulation model. Intensive Care Medicine 39: S222-S223
